# Unraveling the regulatory network of miRNA expression in Potato Y virus-infected of *Nicotiana benthamiana* using integrated small RNA and transcriptome sequencing

**DOI:** 10.3389/fgene.2023.1290466

**Published:** 2024-01-08

**Authors:** Hongping Song, Xinwen Gao, Liyun Song, Yubing Jiao, Lili Shen, Jinguang Yang, Changquan Li, Jun Shang, Hui Wang, Songbai Zhang, Ying Li

**Affiliations:** ^1^ Hubei Engineering Research Center for Pest Forewarning and Management, Yangtze University, Jingzhou, Hubei, China; ^2^ Key Laboratory of Tobacco Pest Monitoring Controlling and Integrated Management, Tobacco Research Institute of Chinese Academy of Agricultural Sciences, Qingdao, China; ^3^ Liupanshui City Company of Guizhou Tobacco Company, Guizhou, Guizhou, China; ^4^ Luoyang City Company of Henan Tobacco Company, Luoyang, Henan, China

**Keywords:** high-throughput sequencing, miRNA, Potato virus Y, miRNA-mRNA network, viral in-fection

## Abstract

Potato virus Y (PVY) disease is a global problem that causes significant damage to crop quality and yield. As traditional chemical control methods are ineffective against PVY, it is crucial to explore new control strategies. MicroRNAs (miRNAs) play a crucial role in plant and animal defense responses to biotic and abiotic stresses. These endogenous miRNAs act as a link between antiviral gene pathways and host immunity. Several miRNAs target plant immune genes and are involved in the virus infection process. In this study, we conducted small RNA sequencing and transcriptome sequencing on healthy and PVY-infected *N. benthamiana* tissues (roots, stems, and leaves). Through bioinformatics analysis, we predicted potential targets of differentially expressed miRNAs using the *N. benthamiana* reference genome and the PVY genome. We then compared the identified differentially expressed mRNAs with the predicted target genes to uncover the complex relationships between miRNAs and their targets. This study successfully constructed a miRNA-mRNA network through the joint analysis of Small RNA sequencing and transcriptome sequencing, which unveiled potential miRNA targets and identified potential binding sites of miRNAs on the PVY genome. This miRNA-mRNA regulatory network suggests the involvement of miRNAs in the virus infection process.

## Graphical Abstract

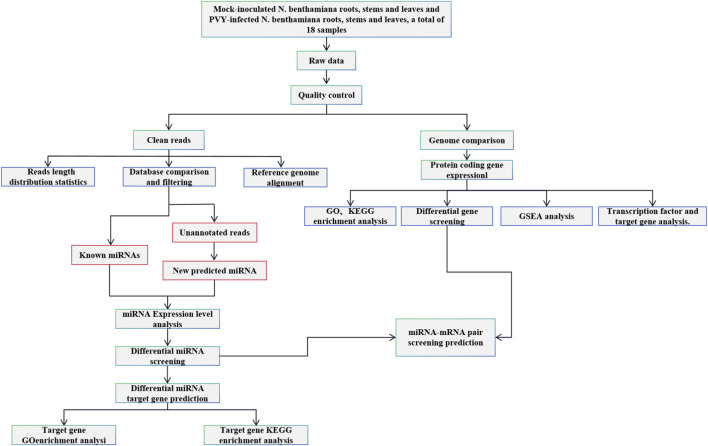

## 1 Introduction

Plant viruses are intracellular parasites and pathogens that consist of nucleic acid, either single- or double-stranded RNA or DNA. They rely entirely on the host cell for replication and dissemination. When a plant is infected by a virus, it can cause disease-like physiological disorders that may lead to plant death. These viral diseases have a significant negative impact on agricultural production, resulting in crop yield losses and posing a threat to global agricultural production (Boualem et al., 2016). Among these pathogens, Potato virus Y (PVY) is particularly detrimental as it causes substantial reductions in both crop quantity and quality (Gray et al., 2010). Persistent PVY infection can lead to a decline in yield ranging from 40% to 60% (Whitworth et al., 2006). Plant RNA viruses, due to their evolvability, large population size, error-prone replication, and strong dependence on host organisms (Qin et al., 2023), have a significant impact on agricultural systems. To date, enhancing crop resistance is the most effective strategy for combating viral infections (Meziadi et al., 2017). Upon viral infection, plants activate a sophisticated antiviral immune response to combat the invader. At the same time, when viruses infect plants, the changes in host genes in different tissues are also different (Naqvi et al., 2011). This may be caused by the different functions of different tissues during virus infection.

MiRNA is the second most abundant sRNA in plants. miRNAs are non-coding RNAs ranging in length from 21 to 24 nucleotides (nt). They function by binding to target mRNA, resulting in translational inhibition or RNA destabilization. As a result, they regulate gene expression at the post-transcriptional level (Davis et al., 2006). Most eukaryotic MIR genes are transcribed by RNA polymerase II (Pol II) to produce primary miRNA transcripts known as pri-miRNAs. These pri-miRNAs have imperfectly folded structures and undergo processing to form stem-loop precursors (pre-miRNAs). The pre-miRNAs are then cleaved to form RNA duplexes. The activation of RNAi occurs through specific double-stranded RNA, which is then incorporated into Argonaute proteins and assembled into RNA-induced silencing complexes (RISCs). These RISCs target mRNA or DNA that is complementary to small RNA (sRNA), thereby exerting regulatory control by binding to and cleaving mRNA, inhibiting translation initiation, and inducing DNA methylation (Ghildiyal and Zomare, 2009; Dang et al., 2011). This intricate process generates various sRNAs, including microRNAs (miRNAs) and small interfering RNAs (siRNAs) (Bologna and Voinnet, 2014). Additionally, miRNAs play significant roles in diverse biological processes, including plant growth, hormone secretion, signaling, protein degradation, and defense against biotic and abiotic stresses (Voinnet, 2009).

Meanwhile, miRNAs play a crucial role in plant-virus interactions (Mengistu and Tenkegna, 2021). To counteract host defense mechanisms, viruses employ various strategies to express viral suppressor genes that inhibit RNA silencing (RSV) during infection (Li et al., 2012; Zhao and Guo, 2022). Conversely, plants activate specific defenses upon viral infection to counteract RNA silencing inhibition (Ding and Voinnet, 2007). Plant miRNAs have the ability to suppress viral expression, induce cleavage of viral mRNA, or impede its translation (Liu SR. et al., 2017; Liu WW. et al., 2017). For example, the endogenous miR393 in plants was the first identified miRNA that enhanced disease resistance by inhibiting the auxin signaling pathway (Navarro et al., 2006). In tobacco plants, miR482 was found to regulate the R gene N of Tobacco mosaic virus (TMV) thereby modulating viral infection by targeting disease-resistant proteins (Shivaprasad et al., 2012; Yang et al., 2015), and overexpression of miR6019 resulted in reduced N transcript accumulation and impaired N-mediated TMV resistance (Li et al., 2012). In previous studies, researchers utilized miR159, miR167b, and miR171 precursors as the basis for designing targeting sequences using artificial miRNA (amiRNA) technology, conferred resistance against PVY and Potato virus X in tobacco plants (Ai et al., 2011). These findings emphasize the critical regulatory role of miRNAs in the viral infection process, exerting direct or indirect control over virus infection dynamics.

Recent investigations have utilized antiviral miRNAs to design and generate amiRNAs, which effectively silence target genes or viruses in plants and achieve robust virus resistance (Duan et al., 2008; Niu et al., 2006; Fahim et al., 2012; Zhang et al., 2018). Therefore, miRNA has great potential as a new type of antiviral small molecule. In this study, sRNA sequencing and transcriptome sequencing were utilized to investigate alterations in miRNA and mRNA levels in tobacco root, stem, and leaf tissues following PVY infection. The research findings reveal the interplay between miRNAs, PVY, and plant endogenous genes, enhancing our comprehension of the host-PVY virus relationship in diverse tissues. Additionally, this study offers novel insights into the potential employment of miRNAs for viral control.

## 2 Results

### 2.1 Analysis of sRNA expression profiles in roots, stems, and leaves of PVY-infected *Nicotiana benthamiana*

**FIGURE 1:**
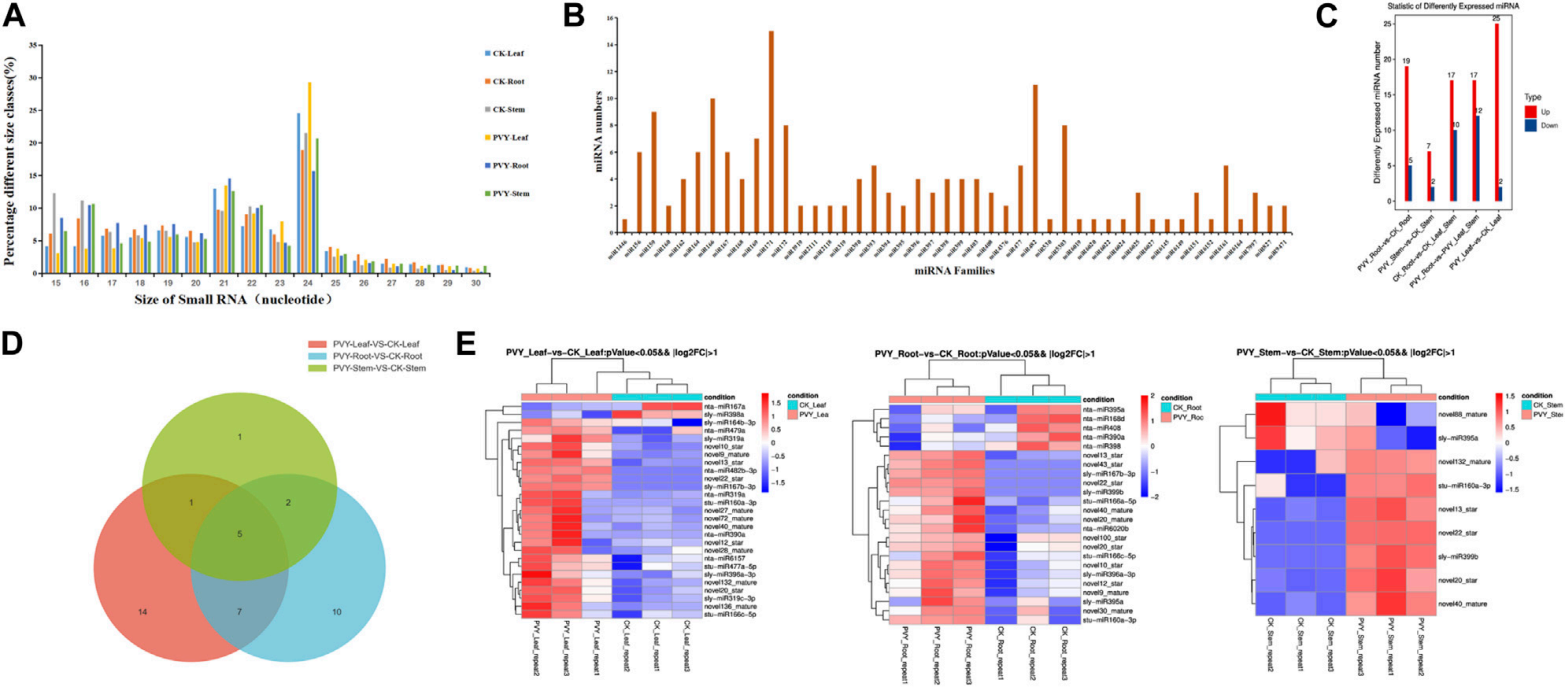
miRNA features of *N. benthamiana* after PVY infection. **(A)** Quantitative distribution of 15-30 nt sRNA in six *N. benthamiana* sequencing samples. **(B)** Quantitative distribution of conserved miRNA families in sequencing data. **(C)** Distribution of differential miRNA numbers. The vertical axis is the number of differentially expressed miRNAs, and Up and Down are the numbers of significantly upregulated and significantly downregulated miRNAs, respectively. **(D)** Venn diagram revealed common significant differences in miRNAs between control and PVY-infected groups of leaves, stems and roots of *N. benthamiana*. **(E)** The heat map shows significantly different miRNAs in *N. benthamiana* roots, stems and leaves under PVY infection (*p* < 0.05). Red indicates higher levels of miRNA and blue indicates lower levels. CK: Control group.

Numerous studies have elucidated the physiological and biochemical changes that occur in plants following viral infection, as plants mount a defense response against viral invasion. sRNAs, particularly miRNAs, have garnered significant attention for their participation in various regulatory pathways governing plant development processes (Umbach and Cullen, 2009). To investigate the alterations in sRNA profiles in plants upon PVY infection, with a focus on miRNAs associated with PVY replication and movement, we generated 18 sRNA libraries derived from PVY-infected *N. benthamiana* (Goodin et al., 2008) roots, stems, and leaves. Supplementary Table S1 provides a summary of the quantities of raw and valid sRNA reads obtained from nine control and nine treatment samples. The results showed that small RNA sizes ranged from 15 to 30 nt, with the most abundant being 21–24 nt as shown in Figure 1A. Following PVY infection, the abundance of sRNAs exhibited diverse changes across the roots, stems, and leaves, with notable variations observed in the size range of 21–24 nt (Figure 1A). The effective reads in the 20–24 nt size category accounted for over 50% of all sRNAs in each sample, with 21 nt and 24 nt reads representing the predominant sRNA types, consistent with findings in dicotyledonous plants (Guo et al., 2017). Notably, we detected a total of 46 miRNA families, although the number of miRNAs varied within each family. The miR171 family exhibited the highest number of miRNAs (15 members), followed by the miR482 family (11 members), with one-third of the miRNA families comprising only one miRNA member (Figure 1B). Among the 46 miRNA families, we identified 110 known miRNA classes and 141 novel miRNA sequences (Supplementary Table S2).

### 2.2 Response pattern of miRNAs in *N. benthamiana* upon PVY infection

To comprehensively investigate the alterations in miRNAs expression in *N. benthamiana* following PVY infection and potentially involved in virus infection of miRNA, we conducted an analysis based on normalized reads obtained miRNAs from high-throughput sequencing. The miRBase database (version 22.0) comprising a vast collection of miRNA mature and hairpin precursor sequences from both plants and animals, was utilized for comparisons. Differential expression analysis was performed by comparing the sequencing data with the miRBase database to identify changes in miRNA expression across the 18 sRNA libraries. In comparison to the control group, the majority (73.28%) of differentially expressed miRNAs showed upregulation in PVY-treated samples. Specifically, we identified 25 upregulated and 2 downregulated miRNAs in *N. benthamiana* leaves, including 12 newly discovered miRNAs and 15 conserved miRNAs. In the stems, we identified 6 novel miRNAs and 3 conserved miRNAs, while in the roots, we identified 11 novel miRNAs and 13 conserved miRNAs (Figure 1C). Among the three treatment groups, only 5 miRNAs displayed significant differences across all three tissues. Additionally, 7 miRNAs exhibited significant differences between leaves and roots, 1 miRNA between leaves and stems, and 2 miRNAs between leaves and roots (Figure 1D). Furthermore, when applying more stringent statistical criteria (fold change >2 and *p*-value ≤0.05), we identified 40 miRNAs with significant differences between the PVY treatment and control groups. Among these, 16 miRNAs showed differential expression in at least one pairwise comparison. The expression patterns of these differentially expressed miRNAs are presented in Figure 1E. Notably, we observed inconsistent expression patterns of the same miRNA across the three tissues. For example, miR390a was significantly upregulated in leaves but downregulated in roots, while miR395a showed significant upregulation in leaves and downregulation in stems. This discrepancy suggests that the regulatory targets of miRNAs may vary among different tissues. The varying expression profiles of miRNAs across PVY-infected tissues indicate potential tissue-specific regulatory roles. These findings highlight the involvement of a complex miRNA-mediated regulatory network in PVY infection, underscoring the need for further investigations to elucidate the precise regulatory roles of miRNAs and identify key miRNAs and target genes associated with PVY infection.

**FIGURE 2:**
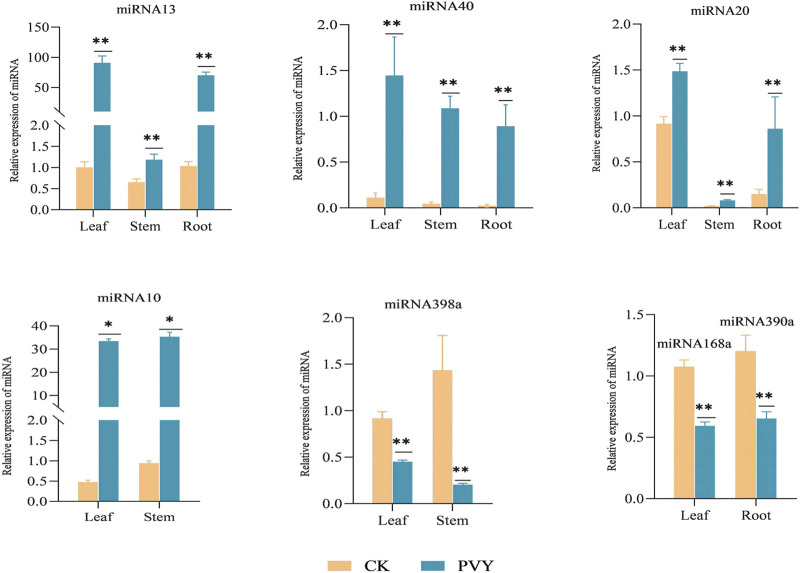
qRT-PCR experiments are conducted to confirm the consistency of the expression levels of specific miRNAs, in line with the sequencing results. The figure displays the relative expression of miRNAs in different tissues of the root, stem, and leaf. The yellow bar represents the healthy *N. benthamiana*, while the blue bar represents the *N. benthamiana* after PVY infection. The expression levels of these miRNAs were determined using stem-loop qRT-PCR, with U6 serving as the loading control. The data shown are the mean plus SD of three biological replicates (n = 3). Statistical significance was determined using Student’s t-test, with *p < 0.05 and **p < 0.01. CK: Control group.

sRNAs were extracted from the samples, and the expression levels of the differentially expressed miRNAs identified through sequencing were assessed using qRT-PCR. Figure 2 presents the qRT-PCR detection results, revealing the upregulation of miRNA13, miRNA40, and miRNA20 in all three tissues following PVY infection, with the most prominent increase observed in the leaf, demonstrating a significant difference. Notably, miRNA10 exhibited significant upregulation in leaves and stems but was undetectable in roots. On the other hand, miRNA398a showed substantial downregulation in both leaves and stems, with a more pronounced reduction observed in the stems. Additionally, the expression levels of miRNA168a and miRNA390a were significantly downregulated in leaves, while their expressions were not detected in roots and stems (Figure 2). Notably, when comparing the qPCR and sequencing results, consistent expression levels of these miRNAs were observed.

### 2.3 Predictive analysis and functional exploration of potential target genes of miRNAs

**FIGURE 3:**
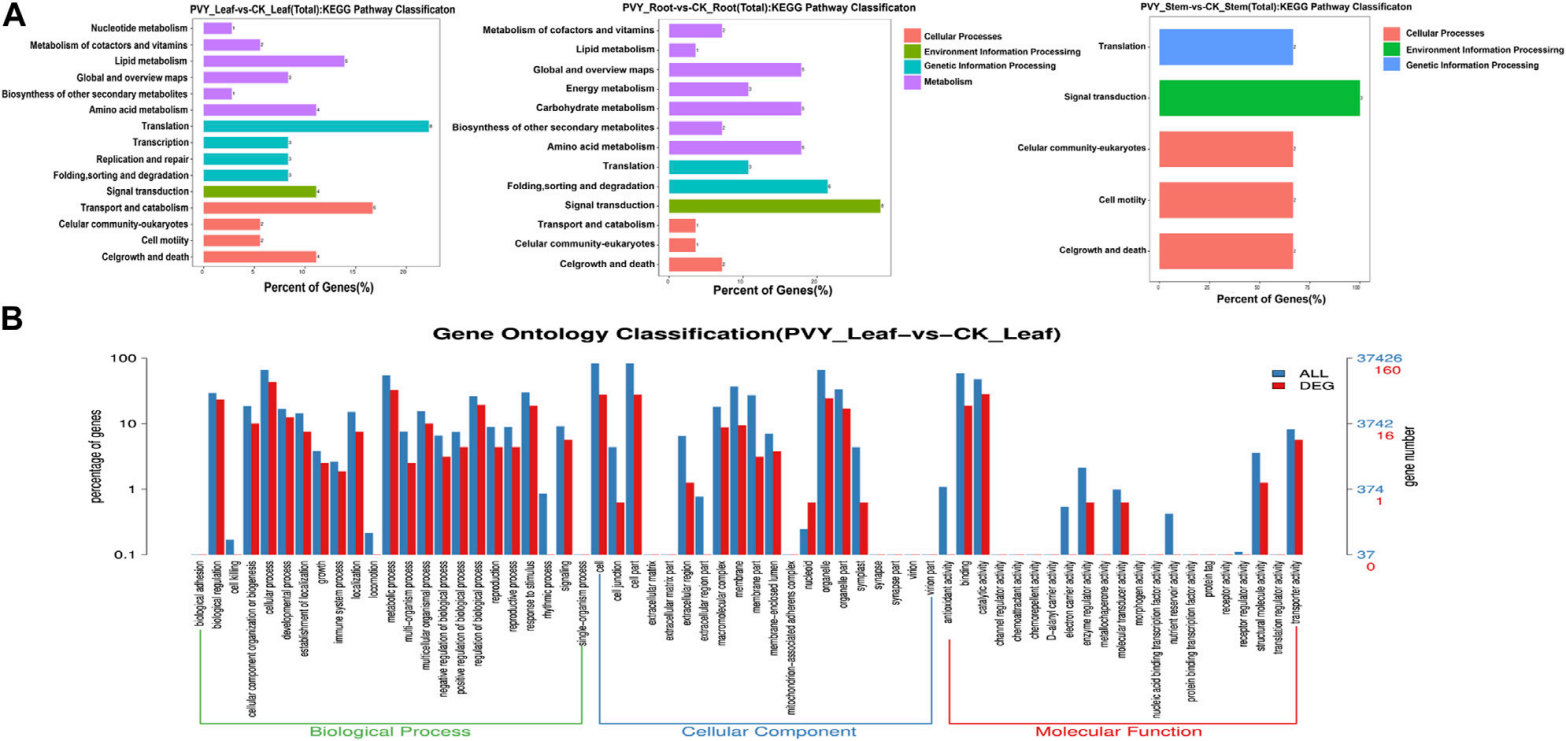
Target gene functions predicted by KEGG and GO enrichment analysis. **(A)** KEGG analysis was conducted on the predicted target genes of miRNA. The functional annotation of KEGG pathways related to the target genes was performed using the KEGG database. **(B)** The functional classification of known miRNA targets was confirmed based on Gene Ontology (GO) categories. The enriched GO data were visualized using WEGO (http://wego.genomics.org.cn/cgibin/wego/index.pl). The left *Y*-axis represents the percentage of predicted target genes in each major category, while the right *Y*-axis represents the number of target genes in each GO category. CK: Control group.

To identify key genes involved in miRNA-mediated interactions during PVY infection, we conducted an analysis of the biological functions of target genes corresponding to differentially expressed miRNAs. The *N. benthamiana* genome served as the target database for the differentially expressed miRNAs obtained through sequencing, and targetfinder software was employed for target gene prediction. The analysis revealed that 39 differentially expressed miRNAs (65.00%) exhibited complementarity to 326 potential target sequences, comprising a total of 354 complementary sites. Notably, over 90% of miRNAs in the miRNA-target gene pairs were predicted to target multiple genes, ranging from 2 to 66, while six miRNAs were predicted to target only one gene (Supplementary Table S3). These miRNA-target gene pairs suggest distinct roles for these miRNAs during PVY infection. Figure 3A dis-plays the diverse array of predicted target genes, encompassing various physiological processes such as growth and development, plant hormones, resistance-related genes, RNA transcription processes, protein-coding genes, enzymes involved in carbohydrate metabolism, and receptor kinases involved in signal transduction (Supplementary Table S4). Among the predicted target genes, miR43 was found to target Auxin-responsive protein IAA29. Additionally, miR10 was implicated in regulating the jasmonate ZIM do-main-containing protein (JAZ), thereby modulating the jasmonate signaling pathway. Moreover, miR390a, miR19a, and miR132 were found to participate in stress response by regulating Growth-regulating factor 3, Auxin response factor 18, and Transcriptional regulator TAC1, respectively. In addition, several receptor kinase genes were identified as target genes for specific miRNAs. For instance, miR390a, miR479a, miR477a, and miR168d were found to target LRR receptor-like serine/threonine-protein kinase (RPK2), Serine/threonine-protein kinase (PPK30), Probable serine/threonine protein kinase IREH1, and Probable serine/threonine-protein kinase PBL23, respectively, indicating the regulatory role of miRNAs in receptor kinases during stress responses. Furthermore, target genes related to the virus were also discovered. For example, miR482 and miR408 were found to target TMV resistance protein N (N), which confers resistance to TMV by mediating defense responses that restrict virus replication and movement (Erickson et al., 1999). Moreover, miR168d was found to target heat shock protein 90-5 (HSP90-5), which is crucial for viral homeostasis and promotes viral replication (Geller et al., 2012). These findings suggest the involvement of these miRNA-target pairs in the viral infection process.

To obtain functional annotations for the differentially expressed miRNAs during PVY infection, we conducted a Gene Ontology (GO) classification n analysis on the predicted miRNA targets. Out of the 326 targets associated with the 39 differential miRNAs, 238 targets were found to possess GO terms (Supplementary Table S5). Among the Biological Process classification, the most prominent categories were cellular process, metabolic process, and biological regulation. Additionally, we performed an analysis of the enriched GO terms among the target genes regulated by the differentially expressed miRNAs. As shown in Supplementary Figure S1, the abundance distribution of the three types exhibited similarities to the patterns observed in Figure 3. Specifically, the leaves and roots displayed similar abundance distributions, while the stems exhibited a relatively lower enrichment of physiological processes.

### 2.4 Integrative analysis of sRNA sequencing and transcriptome sequencing

**FIGURE 4:**
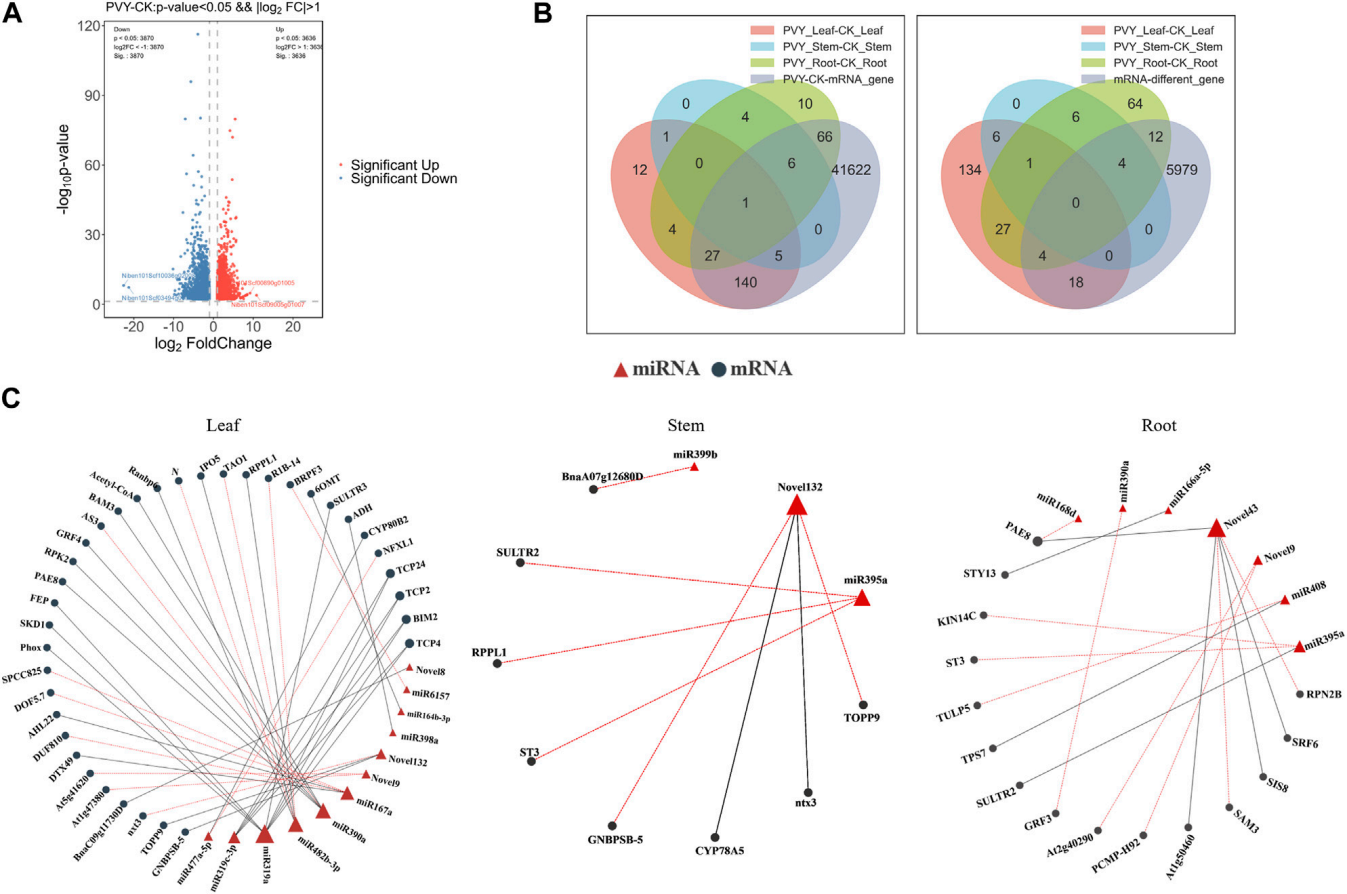
Combined analysis of small RNA sequencing and transcriptome sequencing further clarified the existing miRNA-mRNA pairs and their functions. **(A)** Transcriptome differential gene volcano map. The difference generated by the comparison is reflected in the volcano map, the gray is the non-significant difference gene, and the red and blue are the significant difference gene; the horizontal axis is log2FoldChange, and the vertical axis is log10q-value. **(B)** Venn diagram showing overlap of miRNA target genes with transcriptome total and differentially expressed mRNAs. The left side shows the distribution of the number of differentially expressed mRNAs in roots, stems and leaves, as well as the total differentially expressed mRNAs. The overlapping parts represent shared mRNAs; the right side shows the comparison of the predicted target genes of miRNAs and the differentially expressed mRNAs in the transcriptome. **(C)** miRNA-mRNA network diagram. The relationship between miRNAs and target genes, red triangles represent miRNAs, black circles represent target genes, red lotus lines represent upregulation of target genes, and black lines represent downregulation of target genes. CK: Control group.

To explore the interaction between miRNAs and target genes, transcriptome sequencing analysis was conducted on the 18 samples (Mock-inoculated *N. benthamiana* roots, stems and leaves and PVY-infected *N. benthamiana* roots, stems and leaves, three biological replicates for each sample, a total of 18 samples). A total of 7,506 differentially expressed mRNAs were detected (Figure 4A). Comparison of the predicted target genes with the mRNAs detected in the transcriptome revealed that 245 (87.64%) of the predicted tar-get genes were present in the sequencing data. We compared the predicted target genes of differentially expressed miRNAs detected by sRNA sequencing and the differentially expressed mRNAs detected by transcriptome, and displayed them with a Venn diagram. Further analysis involved comparing the predicted target genes with the differentially expressed mRNAs, resulting in the identification of 36 (13.76%) target genes that matched with the differentially expressed mRNAs (Figure 4B). To gain a more comprehensive understanding of the miRNA-mRNA regulatory network, we constructed a visual representation of the interactions. Notably, the majority of miRNA targets were identified in leaves, with miR319a targeting eight genes, including Phox, SKD1, BIM2, TCP24, TCP2, TCP4, FEP, and SULTR3. Among these, TCP24, TCP4, and BIM2 were targeted simultaneously by multiple miRNAs (Figure 4C). Transcription factors (TFs) play crucial roles in plant defense mechanisms and innate immunity through interactions with cofactors and cisacting elements. The transcriptional regulation of certain TFs can enhance plant defenses against viral infections (Viswanath et al., 2023). Therefore, it is plausible that miRNAs may regulate the viral infection process by targeting TFs. To comprehensively analyze the regulatory network of miRNAs targeting TFs, we performed prediction analysis of transcription factor target genes. The results revealed that the transcription factor ERF had the highest prediction of 18 and 26 target genes in leaves and roots, respectively, while the transcription factor HD-ZIP showed the highest prediction of 23 target genes in stems (Supplementary Figure S2). These variations may arise from the differential distribution of the virus among the three tissues.

Furthermore, to gain deeper insights into the involvement of target genes in physiological processes, we conducted GO and KEGG enrichment analyses analyses using the sequencing data. We observed that the Cellular Component category exhibited the highest level of enrichment in roots, stems, and leaves, whereas the Molecular Function category displayed the least enrichment. Notably, the enrichment patterns across the three tissues were quite similar (Supplementary Figure S3).

### 2.5 Predictive analysis of miRNAs targeting the Potato virus Y genome

PVY, a positive-strand RNA virus, belongs to the Potyvirus genus within the Potyviridae family and possesses a genome length of approximately 9.7 kb. Its genome encodes a large polyprotein that can undergo proteolytic cleavage by virus-specific proteases, resulting in the generation of 10 mature proteins (Karasev and Gray, 2013; Quenouille et al., 2013). These proteins include P1, HC-Pro, P3, 6K1, CI, 6K2, VPg, NIa, NIb, and CP. Previous studies have revealed that miRNAs can exert regulatory effects on virus infection by indirectly modulating key genes involved in physiological processes. Additionally, miRNAs can directly target specific sequences within the viral genome, thereby inhibiting viral gene expression. In light of this, we performed target gene prediction analysis on the differentially expressed miRNAs obtained from the sequencing results and the PVY genome sequence. Our findings revealed that 12 miRNAs were predicted to target specific regions within the PVY genome. Notably, P1 was targeted by miR479a, HC-Pro was targeted by miR482b-3p, P3 was targeted by miR479, anovel10, and miR390a, CI was targeted by miR398a and miR395a, VPg was targeted by miR166a-5p, novel12, novel88, and miR166a-5p, NIa was targeted by novel12, novel88, miR166a-5p, and novel132, NIb was targeted by novel12, miR398a, miR395a, miR6020b, miR6157, and novel30, and CP was targeted by novel88, miR398a, novel43, miR408, and novel22. Remarkably, 8 out of the 10 PVY proteins were found to be targeted by different miRNAs (Table 1). The discovery of miRNAs directly targeting PVY not only aids in the identification of antiviral miRNAs but also offers novel insights for antiviral research.

**TABLE 1:** High-confidence binding sites of miRNAs targeting the PVY genome were predicted by the miranda algorithm.

miRNA	Total energy	Max energy	Positions	miRNA length	Total score
miR479a	−42.58	−17.13	641 3115 2485	22	472
novel88	−40.11	−15.59	6584 6186 8938	19	440
miR398a	−40.11	−16.04	9409 5485 7698	21	439
miR166a-5p	−25.93	−15.68	7062 6512	21	309
novel43	−38.56	−20.29	9322 9535	21	298
miR395a	−29.69	−16.11	3873 7167	21	286
miR6020b	−15.4	−15.4	7700	21	172
miR408	−27.75	−27.75	8901	21	164
miR6157	−20.49	−20.49	7763	22	156
miR482b-3p	−15.01	−15.01	1563	22	154
novel30	−19.5	−19.5	8341	20	152
miR390a	−17.3	−17.3	2473	21	141

### 2.6 Validation of the effects of miR390, miR168, miR32, and miR43 on PVY infection through transient overexpression

**FIGURE 5:**
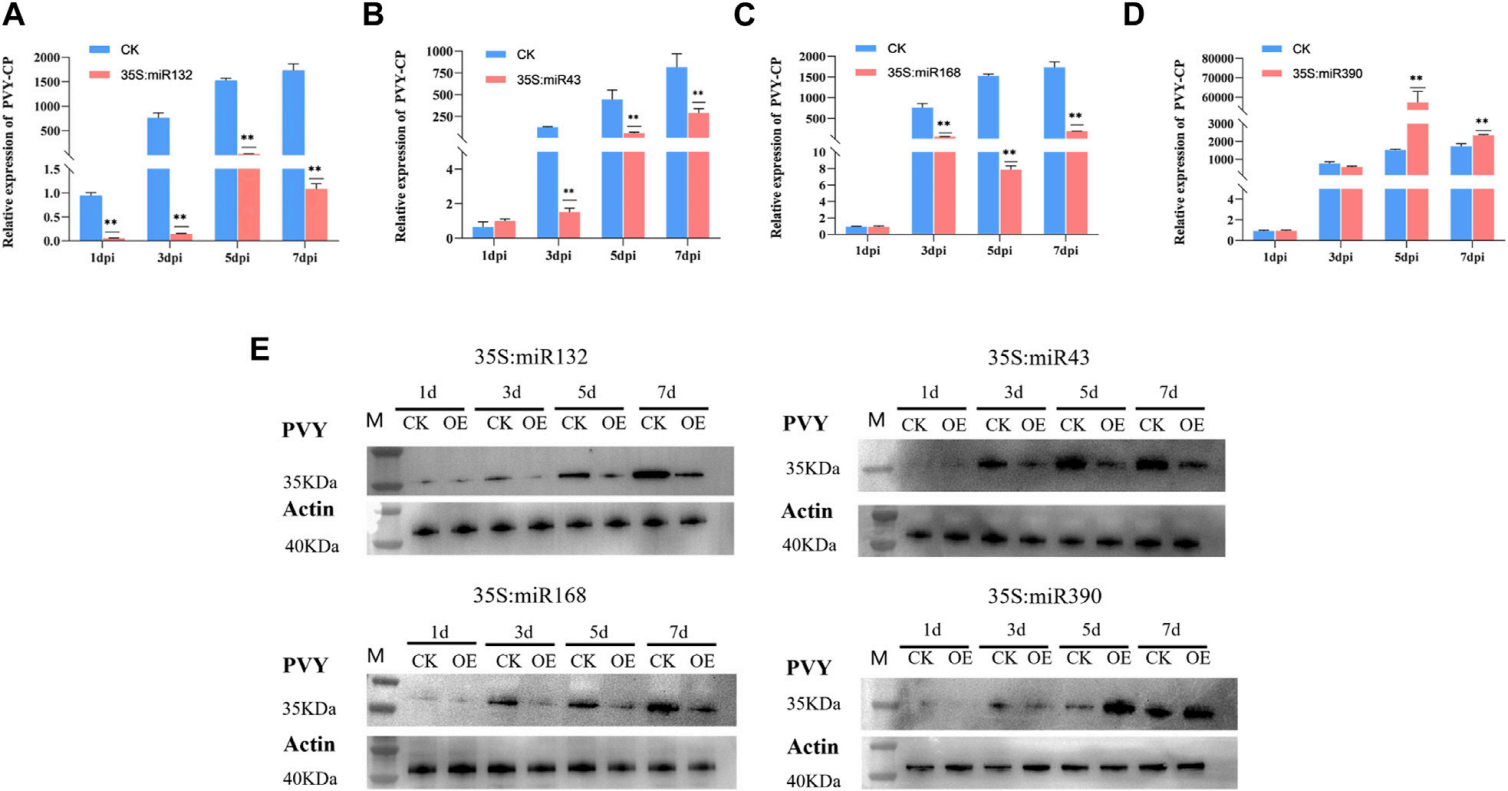
Overexpression of miRNA to verify its effect on PVY infection in *N. benthamiana*. **(A)** Real-time PCR was employed to assess the variations in virus expression at 1, 3, 5, and 7 dpi. The miR132-OE/PVY group was considered as the treatment group, while the WT/PVY group served as the control group. **(B)** Real-time PCR was employed to analyze the variations in virus expression at 1, 3, 5, and 7 dpi. The treatment group consisted of miR43-OE/PVY, while the control group consisted of WT/PVY. **(C)** Real-time PCR was employed to analyze the variations in virus expression at 1, 3, 5, and 7 dpi. The treatment group consisted of miR168-OE/PVY, while the control group consisted of WT/PVY. **(D)** Real-time PCR was utilized to assess the variations in virus expression at 1, 3, 5, and 7 dpi. The treatment group consisted of miR390-OE/PVY, while the control group comprised WT/PVY. **(E)** The expression of PVY CP protein was detected at 1, 3, 5 and 7 dpi. CK: Control group.

**FIGURE 6:**
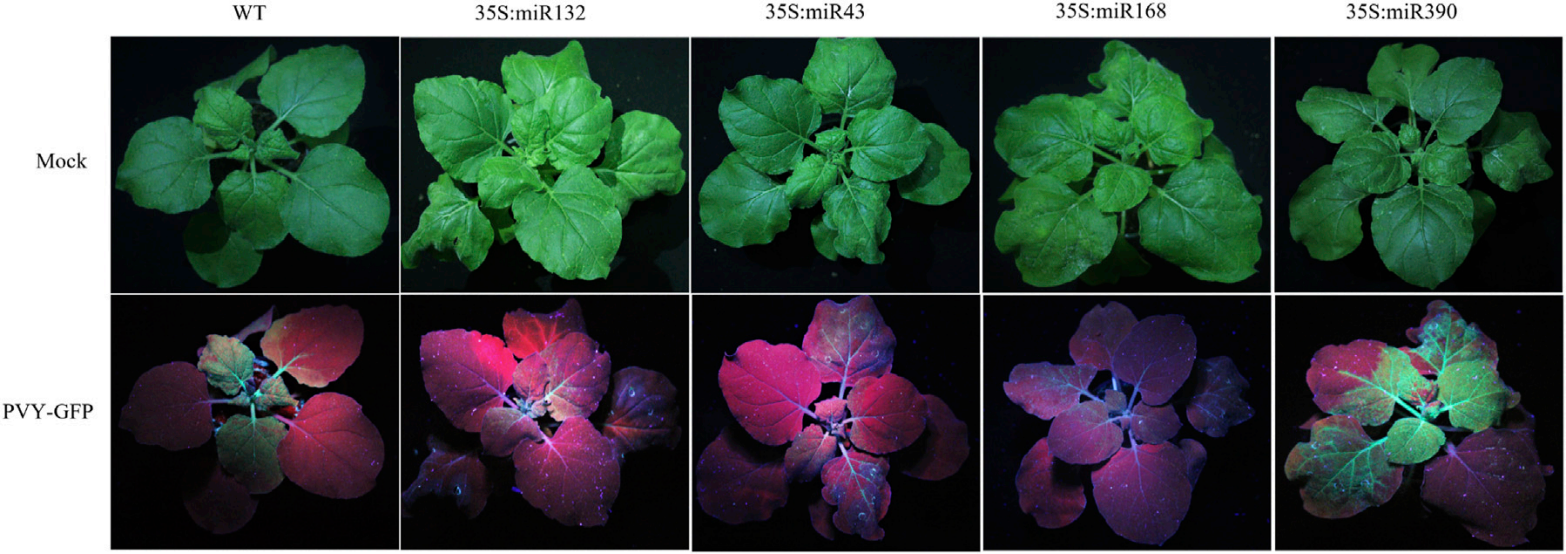
PVY-GFP was used to infect *N. benthamiana* treated with different overexpression of miRMA, and the degree of PVY infection was observed using UV light.

During virus infection, miRNAs exert their regulatory effects by targeting key genes involved in various physiological processes, thereby influencing the progression of the viral infection. To explore the specific role of miRNAs in the context of PVY infection, we selected four differentially expressed miRNAs, namely, miR390, miR168, miR32, and miR43, based on their predicted target genes and functional analysis outcomes. The miR390 family is widely distributed among various plants and plays a role in regulating plant growth and development processes. However, there have been limited studies on its involvement in biotic stress. Previous research has shown that miR168 is involved in the infection process of various viruses. Our attention was drawn to the prediction results of miR43, which targets multiple regulatory genes including AUX/IAA. Additionally, the results of KEGG analysis revealed that miR132 in the stem targets multiple pathways such as MAPK and PI3K-Akt, which we chose to focus on. Overexpression vectors carrying miR390, miR168, miR132, and miR43 were constructed and transformed into Agrobacterium strain LBA4404, followed by introduction into *N. benthamiana* plants previously inoculated with PVY via Agrobacterium-mediated method. In addition, the transcript and protein levels of PVY coat protein (CP) were examined using qRT-PCR and Western blotting, respectively. qRT-PCR was employed to assess the virus expression levels in both the control and treatment groups at 1,3,5and 7 days post-infection (dpi). The results revealed that overexpression of miR132, miR43, and miR168 significantly reduced the transcript levels of PVY-CP in the treatment group compared to the control group at 3 dpi, 5 dpi, and 7 dpi after PVY infection. Notably, miR132 exhibited the most pronounced inhibitory effect on the virus, with inhibition rates exceeding 50% following PVY inoculation. Moreover, the expression of CP protein in the control and treatment groups of *N. benthamiana* showed significant differences (Figures 5A–C). Conversely, plants overexpressing miR390 exhibited significantly increased transcript levels of PVY-CP at 5 dpi and 7 dpi compared to the control group. Notably, the virus increased by more than 10-fold at 5 dpi, and the expression of CP protein in the PVY control and treatment groups displayed notable distinctions (Figure 5D). In this study, we employed PVY-GFP to infect *N. benthamiana* plants that overexpressed four types of miRNA (Figure 6). After 7 days, we observed the fluorescence intensity and phenotype, which were found to be consistent with the Western blotting results. These results demonstrate the regulatory role of miRNAs in modulating PVY infection. To further assess the expression of PVY-CP protein, Western blotting analysis was performed. The results were consistent with the qRT-PCR results, indicating that overexpression of miR132, miR43, and miR168 inhibited PVY infection, whereas overexpression of miR390 promoted viral infection. Taken together, these finZs highlight the regulatory capacity of miRNAs in PVY infection and underline their significance in plant antiviral immunity.

## 3 Discussion

Over the past decade, advancements in high-throughput technologies have greatly facilitated genome and transcriptome sequencing. As a result, there has been a continuous expansion of plant genome and transcriptome databases, which has allowed for the comprehensive identification and functional annotation of plant miRNAs and siRNAs. Several studies have demonstrated that certain miRNAs can play a role in virus infection and enhance a plant’s ability to resist viruses (Lu et al., 2008; Prasad et al., 2019). In this study, we conducted sRNA sequencing and transcriptome sequencing on *N. benthamiana* after PVY infection, and performed a joint analysis of the obtained sequencing results. A total of 60 differentially expressed miRNAs were identified, with 51 upregulated and 9 downregulated. Among these differentially expressed miRNAs, miR168 (Ludman and Fatyol, 2021), miR167 (Liu et al., 2022), miR319 (Zhang et al., 2016), miR164 (Bazzini et al., 2009), miR166 (Prasad et al., 2022), and miR398 (Lin et al., 2022) have been previously associated with virus infection. KEGG enrichment analysis revealed that the predicted targets of miRNAs in the roots, stems, and leaves showed different major enriched signaling pathways. The roots were mainly enriched in PI3K-Akt (Ji and Liu, 2008), Apoptosis (Everett and McFadden, 1999), Wnt (Rim et al., 2022), and Plant hormone (Blazquez et al., 2020) signaling pathways, while the leaves and stems were mainly enriched in RAS, cGMP-PKG, MAPK (Kumar et al., 2018) and other signaling pathways. These signaling pathways are related to viral infection and replication, and the different tissues result in different miRNAs enriching different signaling pathways.

The expression profiles of miRNAs were analyzed after PVY infection in different tissues, revealing inconsistent expression patterns. This inconsistency may be attributed to the ability of miRNAs to target multiple genes and the varying effects of the virus on different tissue physiological processes (Figure 1). By utilizing sRNA and transcriptome sequencing, a potential miRNA-mRNA regulatory network was constructed, as depicted in Figure 4. Through the prediction of miRNA-PVY genome interactions, it was identified that 12 miRNAs target 21 specific sites within the PVY genome (Table 1). The regulatory network revealed the involvement of multiple immune pathways, including the targeting of the tobacco resistance N gene by miR482, which confers tobacco TMV resistance (Dinesh-Kumar and Baker, 2000). Additionally, phytohormones play a crucial role in regulating plant defense responses to various biotic and abiotic stresses, as well as plant growth and development (Huot et al., 2014). This study also discovered several miRNAs that target various plant hormones. MiR43 was predicted to target the key gene AUX/IAA in the auxin pathway, while miR10 targeted the JAZ gene in the JA pathway (jasmonate ZIM domain-containing protein). JAZ is involved in the JA signaling pathway, which regulates plant defense responses (Huang et al., 2022). To evaluate the impact of specific miRNAs on PVY infection, overexpression experiments were conducted on four miRNAs. The results demonstrated that miR168, miR132, and miR43 effectively inhibited virus infection, while miR390 promoted PVY infection (Figure 5). In our study, we discovered that miR168 targets HSP90, which plays a crucial role in viral replication (Verchot, 2012). Additionally, we observed a downregulation of miR168 expression and an increase in HSP90 mRNA expression following PVY infection. Based on these findings, we hypothesized that miR168 targets HSP90 to delay virus replication. Another miRNA, miR390, is predicted to target ACAA1 (acetyl-CoA acyltransferase 1), which is potentially involved in the JA biosynthetic pathway. Previous studies have suggested that miR390 indirectly influences viral infection by regulating plant hormones such as JA and SA (Oka et al., 2013). These studies collectively indicate that miRNA168 and miR390 regulate viral infection through different mechanisms.

Overall, plant-pathogen interactions result in a delicate balance between the plant immune signaling network and the pathogens it encounters. In this study, our focus was on exploring the potential miRNA-mRNA network after PVY infection. We selected four miRNAs for overexpression to assess their impact on PVY infection. This research provides a foundation for the development of effective anti-PVY miRNAs. Our findings demonstrate that the miRNA regulatory network directly affects PVY infection by targeting resistance genes or key genes involved in PVY replication. Moreover, it can also indirectly regulate viral infection by targeting key genes in immune and developmental pathways.

## 4 Materials and methods

### 4.1 Plant materials and viral strains

*N. benthamiana* plants were cultivated in a greenhouse under controlled conditions, with a photoperiod of 16/8 h (light/dark) and a temperature of 25°C. Healthy *N. benthamiana* plants at the 4-week-old stage were subjected to PVY infection or mock inoculation, and tissue samples were collected 7 days post-treatment. Three biological replicates were obtained for each sample. For PVY infection, the infected PVY leaves were diluted 40-fold with phosphate-buffered saline (PBS) and subsequently inoculated onto *N. benthamiana* leaves. Control group (CK): Use 1XPBS to simulate infection on healthy plants. PVY treatment: Use the preserved PVYN:O or PVY-GFP strain to infect *N. benthamiana* (Sun et al., 2018).

### 4.2 Sample preparation

The treatments for *N. benthamiana* are as follows: the control group: using PBS for simulated treatment; the treatment group: using PVY virus to treat the leaves through quartz sand. Sampling was carried out 7 days after treatment, and the roots, stems and leaves of the control group (CK-Root-Repeat1, CK-Root-Repeat2, CK-Root-Repeat3, CK-Stem-Repeat1, CK-Stem-Repeat2, CK-Stem-Repeat3, CK-Leaf-Repeat1, CK-Leaf-Repeat2, CK-Leaf-Repeat3) and the roots, stems and leaves of the treatment group (PVY-Root-Repeat1, PVY-Root-Repeat2, PVY-Root-Repeat3, PVY-Stem-Repeat1, PVY -Stem-Repeat2, PVY-Stem-Repeat3, PVY-Leaf-Repeat1, PVY-Leaf-Repeat2, PVY-Leaf-Repeat3), three biological replicates for each tissue, a total of 18 samples. Then perform sRNA extraction and construct sRNA library.

### 4.3 sRNA library construction and sequencing

sRNAs were extracted from the samples using the Illumina TruSeq Small RNA Preparation Kit (Illumina, San Diego, CA, United States) to construct sRNA libraries. The sRNA library construction involved ligating 10 µg of total RNA from the 18 samples with 3′ and 5′ adapters using T4 RNA ligase. After amplification, 1 µg of the PCR product was subjected to sRNA sequencing on the Agilent 2100 chip platform at Shanghai Ouyi Biotechnology (Shanghai, China) (Billmeier and Xu, 2017). The length and quality of the resulting libraries were con-firmed based on the obtained sequencing results. To identify miRNAs within the data, adapter sequences were initially removed from the original reads using cutadapt (Martin, 2011), and sequences were filtered based on length, excluding those shorter than 15 bp or longer than 41 bp. Subsequently, NGSQCToolkit (version 2.3.2) (Patel and Jain, 2012) was employed to eliminate reads containing N bases, resulting in high-quality clean reads. All valid reads from the 18 sRNA libraries were then aligned to the *N. benthamiana* genome and known plant miRNA sequences. Unannotated sRNA sequences were subjected to prediction using Mirdeep2 (Friedlander et al., 2012) software to identify novel miRNAs, and the secondary struc-tures of novel miRNAs were predicted using RNAfold (Hofacker, 2003) software.

### 4.4 Bioinformatics analysis of sRNA sequences

#### 4.4.1 miRNA expression analysis

Raw reads underwent adapter removal using cutadapt (Kechin et al., 2017), followed by filtering based on length to discard sequences shorter than 15 bp or longer than 41 bp. Quality control was performed using fastx toolkit (version 0.0.13) (http://hannonlab.cshl.edu/fastx_toolkit) software, retaining sequences with a Q20 score of 80% or higher. NGSQCToolkit (version 2.3.2) (Patel and Jain, 2012) was then employed to filter out reads containing N bases, resulting in a collection of high-quality clean reads. The length distribution of these clean reads was statistically analyzed to gain preliminary insights into the distribution of sRNAs within the samples. To determine the proportion of reads aligned to the genome, clean reads were aligned against the reference genome sequence of *N. benthamiana*. Subsequently, clean reads were aligned to the Rfam database using blastn software, enabling annotation of rRNA, snRNA, snoRNA, tRNA, and other sequences. An additional filtering step removed these annotated sequences based on the Rfam database. Moreover, reads matching the transcript sequences with a length less than 15 bp or greater than 41 bp were excluded. RepeatMasker (Chen, 2004) software was utilized to compare the filtered sequences with the repeat database, leading to the identification and removal of repetitive sequences.

#### 4.4.2 miRNA differential analysis

Expression statistics were performed based on the sequences of both known mature miRNAs and the newly predicted miRNAs, with miRNA expression calculated using TPM (transcripts per million) as the metric index. Specifically, TPM (Sun et al., 2014) was determined as the number of reads aligning to each miRNA per sample divided by the total number of aligned reads, multiplied by 106. The significance of differential expression was evaluated using the DEG algorithm within the R package, with miRNAs exhibiting a *p*-value <0.05 considered as differentially expressed.

#### 4.4.3 Differential miRNA target gene prediction and target gene function analysis

Target genes were predicted using miranda and annotated in the reference genome. Target gene prediction for differentially expressed known miRNAs and newly predicted miRNAs was conducted using the targetfinder software. The hypergeometric distribution test of R software was employed for KEGG and GO analyses of the target genes, with the *p*-values subsequently corrected by Benjamini and Hochberg’s multiple tests to obtain the false discovery rate (FDR).

#### 4.4.4 Validation of differentially expressed miRNAs and virus using qRT-PCR

The expression levels of miRNAs identified through sRNA sequencing were verified by qRT-PCR. For expression analysis, we selected six differentially expressed miRNAs and performed qRT-PCR using the 7500 Fast Real-Time PCR System (Applied Biosystems). The forward primer is designed and synthesized based on the specific miRNA sequence, while the reverse primer uses the universal primer Q in the kit (Supplementary Table S6). miRNA extraction from the 18 samples was performed using the EasyPure miRNA Kit (TransGen Biotech, Beijing, China). Subsequently, the extracted RNA underwent reverse transcription using the miR first Strand cDNA synthesis kit (Vazyme, Nanjing, China). The qRT-PCR reaction was conducted on the ABI7500 platform using the 2^−ΔΔCT^ method to calculate the relative expression levels of miRNAs. U6 and actin were utilized as endogenous control and control group1 standard Ct values, respectively. Each experiment consisted of three biological and technical replicates (Song et al., 2023).

#### 4.4.5 Preparation of miRNA expression vectors

To construct the pGate-100-miRNA vector, the miRNA sequence was inserted into the mature miR319a precursor using pRS300 plasmid and specific primers (Ossowski et al., 2008). The miR319a-containing miRNA sequence was then inserted into the pGate-100 expression vector harboring the 35S promoter. The recombinant plasmids were sequenced to confirm correct insertion of the miRNA backbone sequence. Recombinant plasmids were subsequently retransformed into *Escherichia coli* and confirmed by PCR through single colony selection. Purification of recombinant plasmids was accomplished using the Rapid Pure Endogenous Plasmid Maxi Kit (DC202-01; Vazme, Nanjing, China). Plasmid concentration, average optical density (OD) A260/A280, and plasmid purity QA/QC were deter-mined. The target gene was amplified by PCR using pGate-100 universal primers, and the PCR products were subjected to agarose gel electrophoresis, followed by recovery and storage at −20°C.

#### 4.4.6 Western blotting assay

Total protein was extracted from the samples using the Pierce Classic IP kit (Pierce), and protein concentration was determined using the BCA working solution. The protein of interest was separated on a 10% SDS polyacrylamide gel. Immunoblot analysis of PVY-CP was conducted using a rabbit antibody (1:5000, Invitrogen), while viral CP protein was detected using a PVY-CP antibody (1:2000) (Song et al., 2023). The antigens were detected by chemiluminescence using the ECL reagent (SuperSignal West Pico kit) after transmembrane transfer. The membrane was then subjected to fluorescence detection using a fluorescence imager, with β-actin serving as an internal reference for quantitative analysis of band in-tensity. Three independent experiments were performed for analysis.

## Data Availability

The datasets presented in this study can be found in online repositories. The names of the repository/repositories and accession number(s) can be found below: https://www.ncbi.nlm.nih.gov/, PRJNA999491; https://www.ncbi.nlm.nih.gov/, PRJNA991318.

## Supplementary material

SUPPLEMENTARY TABLE S1 sRNA raw read information. The raw image data files obtained by high-throughput sequencing are converted into raw sequencing sequence RawData through base calling analysis; reads containing N bases are filtered out. Finally, high-quality CleanReads are obtained; the number of categories of CleanReads is counted and uniq reads are obtained. CleanReads performs length distribution statistics and counts the repetition frequency of uniq reads.

SUPPLEMENTARY TABLE S2 Among the 46 miRNA families, we identified 110 known miRNA classes and 141 novel miRNA sequences. The number and classification of miRNA families detected in the tissues of tobacco roots, stems and leaves under control treatment and PVY infection; new miRNA sequences detected in sRNA sequencing.

SUPPLEMENTARY TABLE S3 Predicted target gene information of differentially expressed miRNAs in roots, stems and leaves.

SUPPLEMENTARY TABLE S4 Predicted target gene information. Annotation and detailed description of differential miRNAs in roots, stems and leaves in the predicted target genes of Nicotiana benthamiana reference genome.

SUPPLEMENTARY TABLE S5 GO enrichment classification information of target genes predicted by differential miRNAs. GO functional analysis is to functionally annotate and classify all genes and differential miRNA target genes of the species. It includes three major categories: Biological Process (BP) represents the process of a molecular activity event; Cellular Component (CC) represents the cell or its external environment; Molecular Function (MF) It is the active element that describes the gene product at the molecular level.

SUPPLEMENTARY TABLE S6 qRT-PCR Primer Information.

SUPPLEMENTARY FIGURE S1 GO analysis of miRNA putative target genes. GO annotation categorized all of the predicted miRNA target genes and differentially expressed miRNA target genes into biological processes, cellular components, and molecular functions.

SUPPLEMENTARY FIGURE S2 Transcription factor target prediction analysis. The abscissa is the family of transcription factors, and the ordinate is the number of genes. Dark blue represents all genes regulated by this transcription factor family. Orange represents the differential transcription factors regulated by this transcription factor family. Pink represents the differential transcription factors regulated by this transcription factor family with an upregulation trend. Light blue represents differential transcription factors regulated by this transcription factor family with a trend of downregulation.

SUPPLEMENTARY FIGURE S3 GO analysis of differentially expressed mRNAs. The first circle: the classification of enrichment, and the scale outside the circle is the number of genes. Different colors represent different classifications; the second circle: the number and Q value or p-value of the classification in the background genes. The more genes, the longer the bar, the smaller the value, the redder the color, and the larger the bluer; the third circle: the bar graph of the proportion of upregulated genes, light red represents the proportion of upregulated genes, light blue represents the proportion of downregulated genes; the bottom shows the specific Value; the fourth circle: the RichFactor value of each category, and each cell of the background auxiliary line represents 0.2.
