# Supplementary figures and images for: Unraveling the regulatory network of miRNA expression in Potato Y virus-infected of Nicotiana benthamiana using integrated small RNA and transcriptome sequencing
Source: Front Genet. 2024 Jan 8;14:1290466. doi: 10.3389/fgene.2023.1290466 (PMC10800900; doi:10.3389/fgene.2023.1290466)

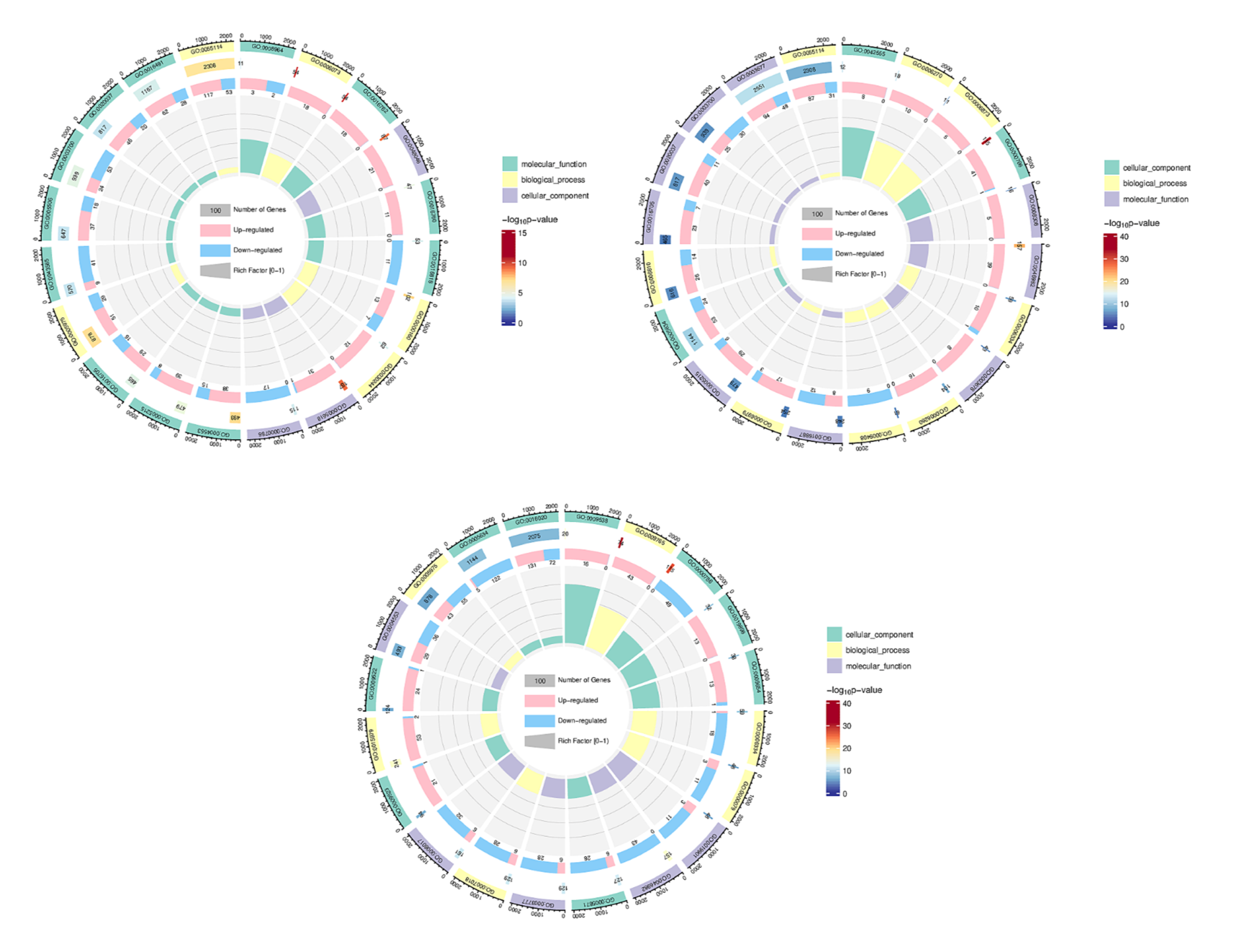

Supplement: Supplementary file 3 [file Image3.TIF]

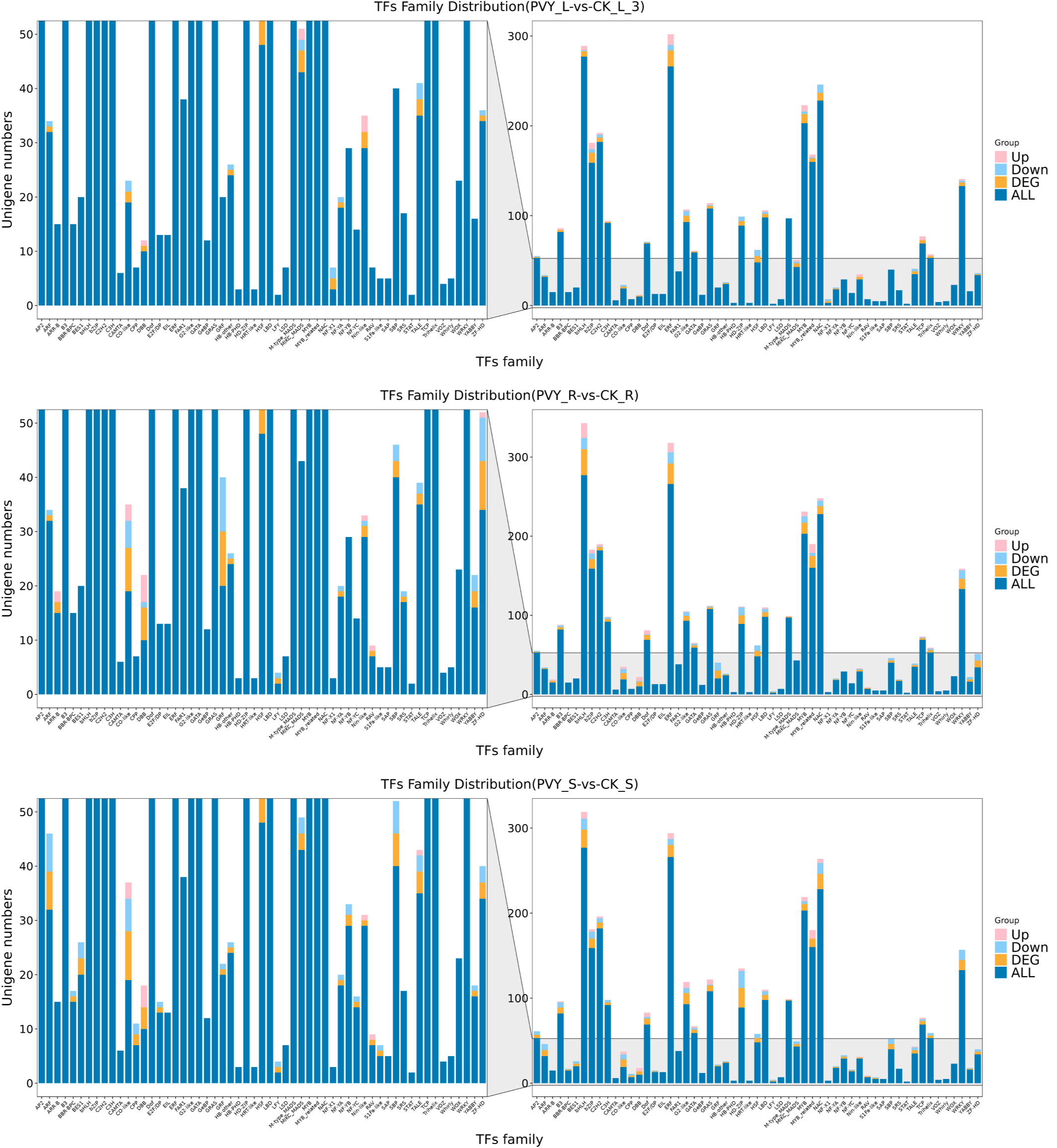

Supplement: Supplementary file 4 [file Image2.TIF]

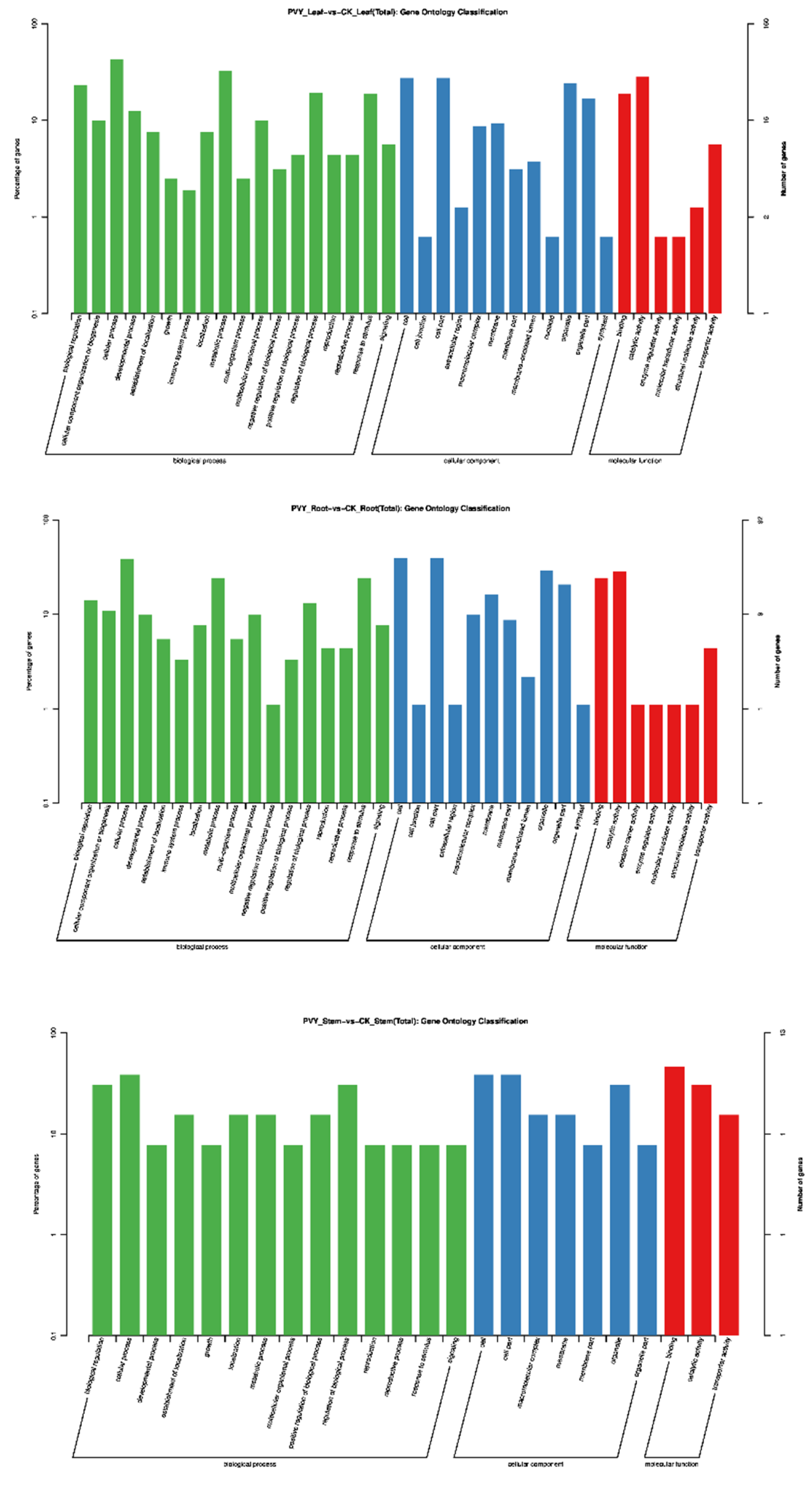

Supplement: Supplementary file 5 [file Image1.TIF]

Western blot

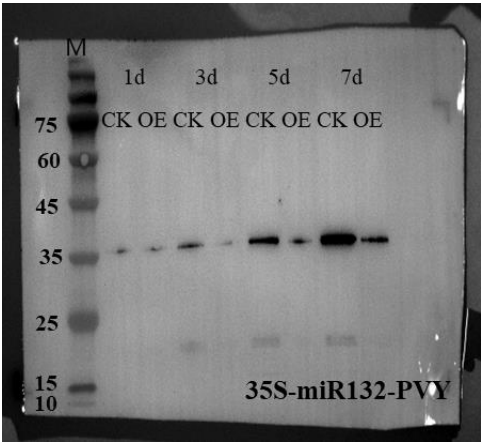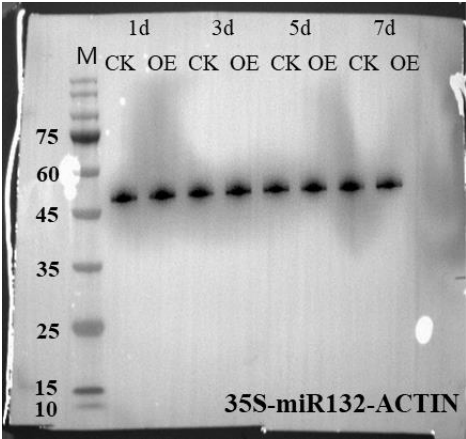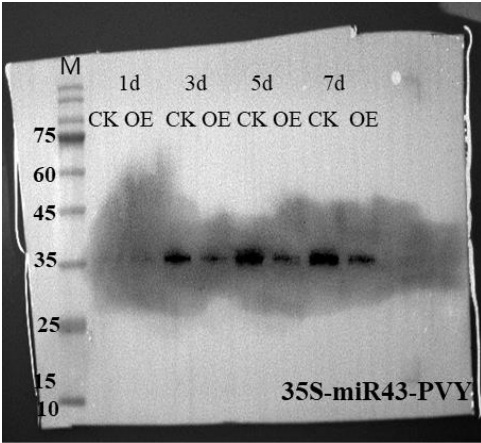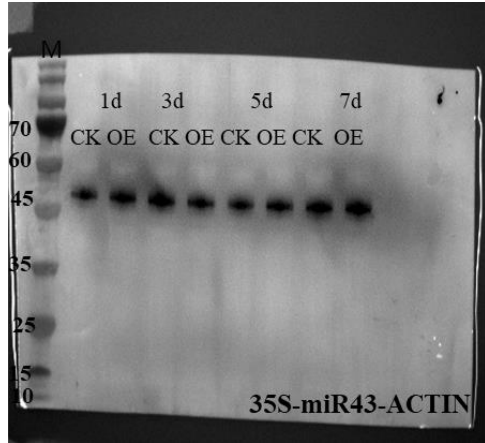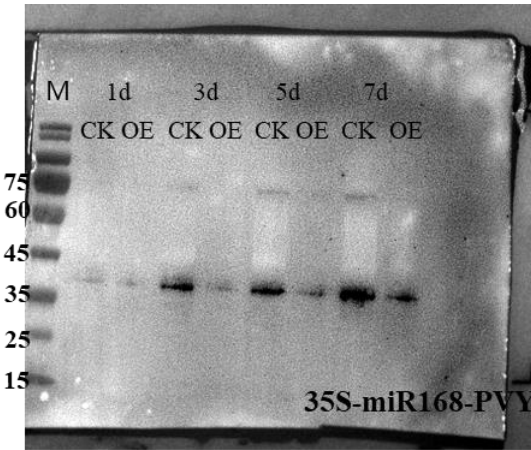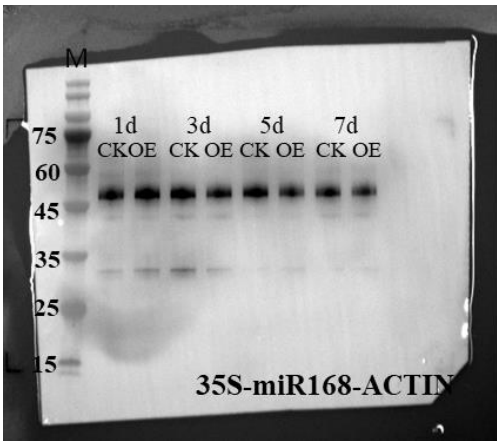

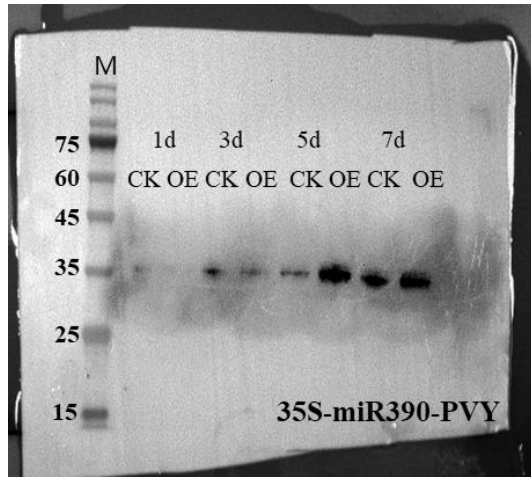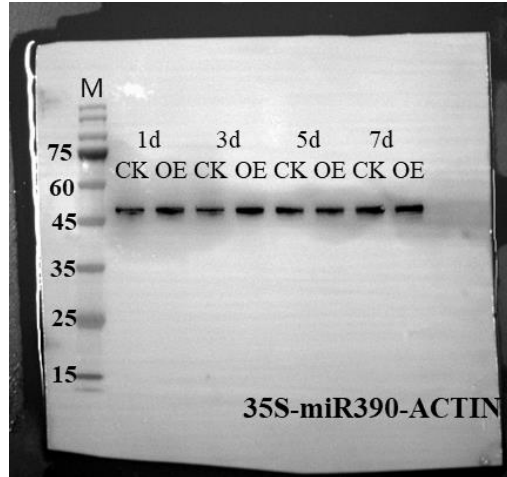

Supplement: Supplementary file 6 [file Image4.pdf]
